# Senescent macrophages in tumor: phenotypes, roles, and interventions

**DOI:** 10.1038/s41419-025-08000-5

**Published:** 2025-10-06

**Authors:** Wenhui Shen, Yueyu Huang, Xuping Yang, Yutian Zhang, Yiyi Pan, You Xiao, Jiahui Wang, Changchun Wang, Weimin Mao, An Zhao

**Affiliations:** 1 Zhejiang Cancer Institute, Zhejiang Cancer Hospital, Hangzhou Institute of Medicine (HIM), Chinese Academy of Sciences, Hangzhou, Zhejiang China; 2 Department of Thoracic Oncology, Zhejiang Cancer Hospital, Hangzhou Institute of Medicine (HIM), Chinese Academy of Sciences, Hangzhou, Zhejiang China; 3 Thoracic Oncology Laboratory, Jiangxi Cancer Hospital, Nanchang Medical College, Nanchang, Jiangxi China

**Keywords:** Senescence, Cancer microenvironment

## Abstract

The senescence of immune cells, including macrophages, that accompany the initiation and development of tumors has become a novel research hotspot. Recently, studies have reported the molecular characteristics of senescent macrophages (sMACs) in the tumor microenvironment (TME), including cell cycle arrest, senescence-associated secretory phenotype (SASP), and senescence-associated β-galactosidase phenotype (SA-β-gal), and these characteristics not only suggest that sMACs are functionally rich in the TME, but also have the potential to become biomarkers for the identification of sMACs. The in-depth study and analysis of sMACs dialogue and mediating the changes of signaling pathways related to tumor and immune cells will help us to better understand the balance between tumor and aging. Here, we review recent advances in sMACs, including phenotypical molecular characteristics, potential functions and intervention approaches.

## Facts

- Senescent macrophages are a component of the tumor immune microenvironment and play a multifaceted role.
- Senescent macrophages may exhibit molecular differences in various tumors, and their identification requires the use of single-cell omics or combinatorial biomarker strategies.
- Senescent macrophages regulate the infiltration and function of immune cells in tumors by secreting factors such as SASP or by expressing proteins like PDL1.
- Targeting senescent macrophages is a novel and promising therapeutic strategy in oncology.

## Open questions

- How to establish a reliable methodological framework to achieve accurate qualitative and quantitative assessment of senescent macrophages?
- What are the detailed molecular mechanisms of senescent macrophages in promoting tumor initiation, progression, and immunomodulation?
- How to develop new therapeutic strategies based on senescent macrophage-specific targets to achieve effective antitumor responses?

## Introduction

Senescence has been recognized as one of the novel hallmarks of cancer. Cellular senescence occurs when proliferating cells enter a stable state of cell cycle arrest under conditions of damage or stress. This state encompasses a range of physiological processes and is intricately linked to tumors, Alzheimer’s disease, and other age-related diseases (ARDs) [1–4]. The deficiency of endogenous telomerase activity and telomere attrition are fundamental factors that contribute to cellular dysfunction and senescence. Hallmarks of senescence include irreversible cell cycle arrest, buildup of DNA damage, enhanced lysosomal activity, and metabolic dysregulation linked to mitochondrial impairment, including alterations in silent information regulator and mechanistic target of rapamycin (mTOR) signaling pathways [2, 3, 5, 6]. Abnormal cytokines or chemokines are secreted by senescent cells, collectively termed the senescence-associated secretory phenotype (SASP), which plays a critical role in immune-cell communication [3, 7–9]. SASP refers to the pro-inflammatory molecules secreted by senescent cells. While some investigations proposed that cellular senescence facilitates angiogenesis, tissue healing, and suppression of tumor growth [10–12], accumulating evidence reveals that, during chronic injury, senescent cells can evade immune surveillance by secreting SASP. This evasion fosters the accumulation of senescent cells, which disrupts the tissue microenvironment and may contribute to the onset of various ARDs, including malignancies [1, 7, 10, 13].

The tumor microenvironment (TME) refers to the local region in which tumor cells reside, incorporating a diverse array of non-tumor cell types, extracellular matrix (ECM) components, vascular networks, and soluble factors. This intricate milieu is critical in modulating tumor behavior and influencing therapeutic responses [14, 15]. TME-associated senescent cells manifest a dualistic role: on the one hand, senescence TME statue can inhibit tumor progression by slowing their proliferation [11]; conversely, the dynamic interplay of immune cell functions and cytokines produced by senescent cells allows these senescence-associated factors to modify the immune escape mechanisms within the TME, thereby significantly promoting tumor growth and the spread of cancer to distant sites [7, 10, 16, 17].

**Fig. 1:**
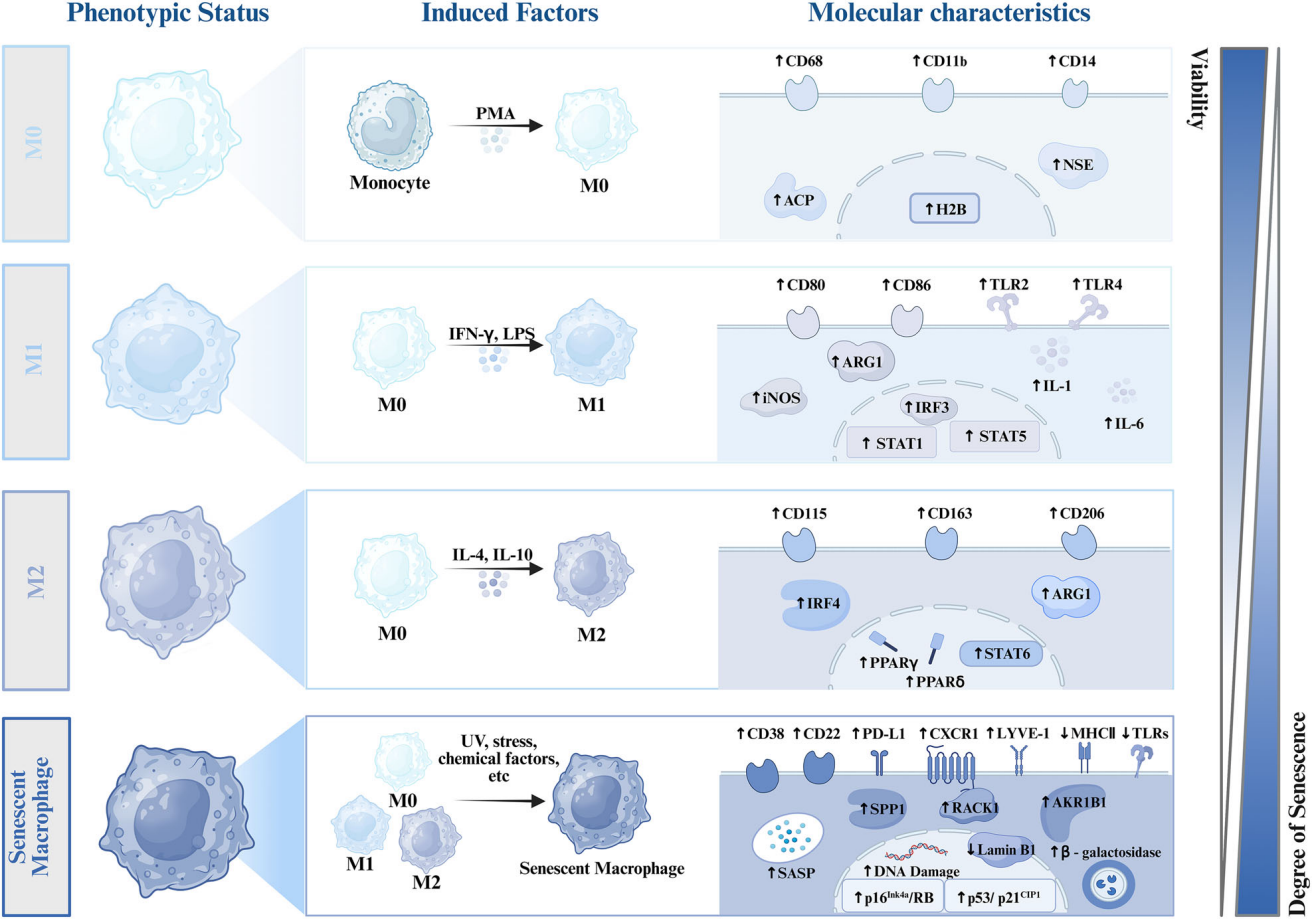
Inducing factors and related molecular characteristics of different macrophage phenotypes. M0 macrophages are derived from monocytes under the stimulation of PMA. M0 macrophages can differentiate into M1 macrophages upon induction by IFN-γ and LPS or M2 macrophages under the influence of IL-4 and IL-10, accompanied by corresponding molecular characteristic changes. Under stimulation by factors such as UV radiation, stress, or specific chemical agents, macrophages can transition into senescent macrophages, exhibiting general senescence-associated phenotypic changes such as increased expression of P16 and P53, elevated secretion of SASP, increased β-galactosidase activity, enhanced DNA damage in the nucleus, and reduced Lamin B expression. Additionally, they display specific markers of senescent macrophages, including elevated expression of CD22, CD38, PD-L1, CXCR1, RACK-1, LYVE-1, and AKR1B1, as well as decreased expression of MHC II and TLRs. The polarization process from M0 macrophages to senescent macrophages is characterized by a gradual decline in viability and a progressive increase in the degree of senescence.

Macrophages are essential components of innate and adaptive immunity and are one of the major infiltrating immune cells in TME. These macrophages are known as tumor-associated macrophages (TAMs) and play a dual role in tumor initiation and progression, acting as promoters and tumorigenesis suppressors [18, 19]. Macrophages can be classified into two distinct subtypes, also known as the polarization states of macrophages: M1 type and M2 type (Fig. 1). M1 macrophages are classically triggered, pro-inflammatory cells that exert direct tumor-suppressive effects by secreting pro-inflammatory cytokines, such as interleukin-6 (IL-6), and generating reactive oxygen species and reactive nitrogen species, all of which contribute to the amplification of anti-tumor immune responses. In contrast, M2 macrophages release immunosuppressive mediators, including IL-4, IL-10, and transforming growth factor-beta (TGF-β), which inhibit T cell and natural killer (NK) cell functionality, promote angiogenesis, and facilitate tumor cell invasion, thereby contributing to tumor progression [19–21].

Contrary to the disordered proliferation of tumor cells, immune cells such as macrophages present a state of exhaustion or senescence-related reprogramming in TME. In 1978, Johnson et al. noted a significant reduction in the cytoplasmic spreading ability of macrophages in aged mice [22]. Subsequently, several studies have established that, as aging progresses, macrophages exhibit a progressive decline in phagocytic capacity, respiratory burst activity, levels of toll-like receptors (TLRs) and MHC class II (MHC-II), responsiveness to antigenic triggers, and the release of pro-inflammatory chemokines and cytokines [23–28], suggesting that the senescence of macrophages occurs with aging, and the molecular changes of senescent macrophages (sMACs) disrupt the regular immune cell dialog. In addition, in vivo animal experiments showed that elimination of sMACs could inhibit tumor growth, suggesting the clinical significance of combination therapy targeting sMACs [29, 30]. Nonetheless, there is still a lack of consensus regarding the characteristic phenotypes of sMACs within the TME and their mechanistic roles. This review aims to provide an overview of the current understanding of the tumor infiltration-sMACs, summarize the molecular characteristics and functional abnormalities of sMACs, and discuss the potential roles and interventions of sMACs.

## Molecular characteristics of senescent macrophages (sMACs)

Senescent cells may occur at any stage of life, and real-time quantitative detection of cellular senescence is an important prerequisite for research. In 2019, the International Cell Senescence Association established an extensive definition of cellular senescence, outlined its hallmark characteristics, and recommended detection approaches, emphasizing the necessity for a multifaceted assessment that integrates various senescence markers to identify senescent cells accurately [3]. But, sMACs is still not well defined and guideline biomarkers are lacking. Evidence suggests that, like most senescent cells, sMACs secrete mediators such as IL-6 and TNF that enhance a proinflammatory phenotype. Additionally, sMACs show increased expression of inflammatory cytokine genes following TLRs activation, likely resulting from the reduced ability of sMACs to downregulate TLRs signaling pathways [31–34]. Hall et al. demonstrated a marked elevation in p16 and β-galactosidase levels in senescent M2 macrophages, supporting earlier hypotheses regarding the potential phenotypic resemblance linking senescent cells to M2 macrophages [35]. Crystal et al. found that chemotherapy-induced senescent tumor cells acquire a survival advantage by engulfing neighboring cells, exhibiting gene expression changes associated with phagocytic function [36]. Additional research has indicated that macrophages may undergo cell cycle arrest simultaneously with acquiring a pro-inflammatory phenotype, leading to the term “senescence-like macrophages” [37, 38]. The above observations highlight an aging polarized state or senescence of macrophages distinct from M1/M2 polarization. Collectively, the differentiation factors and specific molecules of sMACs are significantly different from those of M1-type and M2-type macrophages (Fig. 1).

### Cell cycle-related genes

Cell cycle arrest, mainly governed by the p16^Ink4a^/RB and p53/p21^CIP1^ signaling pathways, represents a fundamental characteristic of senescent cells [39, 40]. A strong correlation has been demonstrated between p16 expression at both the RNA and protein levels and the state of cellular senescence [4]. Cellular senescence is regarded as a permanent state of arrest that cannot be reversed by known physiological stimuli [41]. Grosse et al. developed a knock-in mouse model, which specifically targets p16^Ink4a^ and identified a population of macrophages in these mice with significantly increased of p16^Ink4a^ expression [38]. Liu et al. further observed that, as aging progresses and inflammation accumulates, the subpopulation of macrophages with elevated p16^Ink4a^ levels gradually increased, accompanied by the upregulation of molecules indicative of senescence [42]. Moreover, in vitro experiments utilizing oxidized low-density lipoprotein (Ox-LDL) to induce senescence in RAW264.7 mouse macrophage cell lines demonstrated significant upregulation of p53, p21, and p16 expression levels, suggesting that macrophage senescence also presents senescence-associated phenotypic characteristics [43]. Hall et al. found that tissue macrophages can express high p16^Ink4a^ expression when subjected to alginate encapsulation, with the associated increases in senescence-associated β-galactosidase (SA-β-gal) and p16^Ink4a^ levels under these conditions being reversible. These macrophages remain responsive to polarization stimuli, suggesting that high expression of p16^Ink4a^ in macrophages does not necessarily indicate senescence but instead represent a physiological adaption to immune activation [35]. Milan R. et al. observed that mouse microglia exhibit shortened granules and markers of DNA damage after multiple passages, but such features were not detected in microglia from aged mice. Additionally, the low p16 expression found in both early-passage microglia and those isolated from 3-month-old mice further indicate p16 may not serve as a definitive marker for macrophage senescence [44]. PMA-induced macrophages derived from THP-1 cells in vitro exhibit cell cycle arrest in the G2 or M phase [45]. Human macrophages stimulated by the TLR4 agonist, lipopolysaccharide (LPS), exhibit a secretory pro-inflammatory phenotype accompanied by cell cycle arrest [46]. These results suggest cell cycle arrest may be a primary characteristic of terminally differentiated macrophages [37]. While it remains unclear whether cell cycle arrest definitively signifies sMACs, current recommendations suggest evaluating macrophage senescence status by combining the analysis of p16 or p21 alongside additional relevant senescence markers.

### Lysosomal-senescence-associated β-galactosidase axis

One of the typical features of senescent cells, including sMACs, is the expansion of the lysosomal compartment, leading to significantly enhanced β-galactosidase activity [47–49]. Although this marker can also be expressed in other cellular physiological processes, SA-β-gal has become one of the most commonly used methods for detecting cellular senescence due to its simplicity and relatively high specificity [3, 6]. However, an increasing body of evidence suggests that normal macrophages may also demonstrate enhanced lysosomal capacity. Kopp et al. reported the detection of SA-β-galactosidase-positive expression in osteoclasts across various age stages in mice, which may result in misleading background interpretations [50]. This phenomenon has similarly been observed in the liver’s brain microglia and Kupffer cells, indicating that lysosomal expansion represents a key characteristic of macrophages [51, 52]. Likewise, the differentiation of monocytes into macrophages is predominantly marked by an increase in the quantity of lysosomes [45]. Moreover, several studies have shown that macrophages exhibit a marked increase in SA-β-gal expression under stimulatory conditions, such as radiation, accompanied by additional senescent phenotypes [53, 54]. There was a significant negative correlation between the expression of KI67 and SA-β-gal in expanded culture macrophages in vitro [55]. Additionally, another report noted that in the tissues of older mice, most cells co-expressing SA-β-gal and p16 were macrophages [56], suggesting the potential relevance of SA-β-gal for studies on sMACs.

Currently, a standardized protocol for staining β-galactosidase in sMACs is lacking. Moreover, a key lysosomal characteristic linked to cellular senescence is the accumulation of lipofuscin, which can be effectively detected using Sudan Black B (SBB) staining and utilized in paraffin-embedded tissue sections. Compared to SA-β-galactosidase staining, SBB staining is more widely applicable but demonstrates lower specificity [57]. In addition. Factors such as temperature and fixative solution during specimen processing may lead to the inactivation of SA-β-gal, which is the difficulty for its stable detection.

### Senescence-associated secretory phenotype

The collection of cytokines (IL-1a, IL-6), chemokines (CXCL12), growth factors (FGF, VEGF), matrix metalloproteinases (MMPs), and regulatory factors like TGF-β are collectively known as SASP [2, 58–60]. Studies demonstrated that both sMACs and other senescent cells can secrete SASP, and its plays a crucial role in sustaining chronic, systemic, low-grade inflammation and exacerbating the dysregulation of inflammatory responses during aging. This contributes to the pathogenesis and progression of various ARDs, including tumor [61]. Serval studies have elucidated the significant roles of senescent cells in the initiation and progression of tumors [62]. The earliest evidence demonstrating the paracrine tumorigenic activity of senescent cells was observed in senescent fibroblasts [63]. Senescent stromal cells are known to release elevated amounts of MMP-1, MMP-3, and MMP-10, which support tumorigenesis by remodeling the ECM [4]. In addition, senescent cells secrete the SASP, notably IL-6, which upregulates HLA-E expression, thereby suppressing the immune activity of NK cells and T cells against senescent cells [64]. By releasing IL-6 and IL-8, the SASP promotes the development of precancerous chronic inflammation, facilitating epithelial-mesenchymal transition (EMT) [65]. Furthermore, the SASP can recruit myeloid-derived suppressor cells (MDSCs) by releasing various cytokines, thereby exacerbating the inhibition of anti-tumor immune responses [66].

Recently, by performing single-cell sequencing of thymoma tissues, we identified a macrophage subset expressing CCL3^+^, indicating the possible presence of sMACs that secrete the SASP within the TME [67]. Research suggests that LPS-induced sMACs exhibit significantly elevated levels of pro-inflammatory factors, such as TNF-α, IL-6, COX-2, and PGE2 [46, 68]. Scott et al. employed a novel P16-FDR mouse model, revealing that macrophages exhibiting senescent features express a distinct array of pro-tumor SASP factors, including BMP2, IL-10, CCL2, CCL7, CCL8, and CXCL13, which facilitate lung cancer progression by activating the KRAS signaling pathway [69]. Notably, SASP-associated CCL7 and IL-10 have been demonstrated to significantly promote tumor cell invasion and metastasis [70–72]. Liu et al. discovered a population of macrophages in the aging liver that expresses CXCL2 and possess the capacity to secrete SASP factors; these sMACs utilize the CXCL2-CXCR2 axis to attract neutrophils, which in turn further stimulate the formation of neutrophil extracellular traps (NETs) by secreting IL-1β and TNF-α; the pathological accumulation of NETs is associated with liver damage, which may act as an early event in the development of hepatocellular carcinoma [73–75].

## Discovery and identification strategies of sMACs

### Discovery of sMACs by single-cell multi-omics

The rapid development of single-cell multi-omics technology in recent years has enriched our understanding of the identification of cell subpopulations in tissues and their evolutionary relationships [76]. By constructing a single-cell atlas of macrophages and performing unsupervised clustering analysis, sMACs with aging characteristics can be highlighted from the single-cell atlas [77].

Brittany et al. performed single-cell transcriptomic analysis of healthy breast tissue and found significant age-related expansion of myeloid cell populations, particularly M2-like macrophages. These cells progressively accumulated during aging and exhibited high expression of chemokines CCL7 and CCL8 at the transcriptomic level [78]. As CCL8 is a canonical component of the SASP, this subpopulation likely possesses senescent features and may represent a functionally distinct population of sMACs involved in shaping the aging tissue microenvironment [79]. Matthew et al. used single-cell and epigenetic analysis of an aged mouse lung cancer model and found that the age-related decline in DNMT3A expression in aged myeloid cells activates IL-1α expression, thereby promoting tumorigenesis, suggesting a potential mechanism of aging-related myeloid cell population in carcinogenesi [80].

Additionally, recent advances in technologies such as single-cell live imaging combined with mass spectrometry (SCLIMS) enable simultaneous acquisition of cellular metabolic profiles and phenotypic states, offering an innovative tool for studying metabolic reprogramming during cellular senescence, with promising applications in sMACs research [81].

### Combining biomarkers to identify sMACs

The omics screening technology improves the discovery probability of sMACs, but the qualitative and quantitative examination of the distribution observation in the tissue is key to carrying out in-depth research. Considering the cell species and senescence characteristics of sMACs, such as macrophage markers (e.g., CD14 and CD68) and senescence signatures (e.g., P16, SASP factors, and lysosomal markers), a combination of at least two of the above biomarkers is recommended for the identification of sMACs. Therefore, the sMACs marked with multiple fluorescence can be detected by multiple immunofluorescence or flow cytometry, and the quantitative data of sMACs can be obtained by automatic calculation of software or algorithms. In addition, M1-type or M2-type macrophage markers could also be designed in the experiment for co-staining to increase the reliability of obtaining the sMAC ratio from the perspective of negative fluorescence.

## Phenotypic and functional abnormalities of sMACs

As various physiological functions decline with human aging, sMACs also manifests as a decline in multiple functions, including phagocytosis, oxidative stress resistance, cytokine secretion, tissue repair, autophagy, respiratory capacity, and the expression of TLRs and MHC-II molecules [27, 82].

Macrophages, essential components of the innate immune cells, are highly efficient at engulfing and eliminating pathogens, playing a crucial role in the immune defense. Research shows that when cultured for extended periods, sMACs significantly decrease their phagocytic capacity and produce fewer inflammatory cytokines when stimulated with LPS [55]. Yohko et al. demonstrated that the impaired activity of the aging-associated gene P53 plays a pivotal role in the reduced phagocytic capacity of macrophages in aged mice. This highlights that aging directly affects the ability of macrophages to perform phagocytosis [83]. Several animal studies further validate this idea, revealing that macrophages in aged mice have a diminished capacity for phagocytosis, leading to increased vulnerability to diseases like influenza [84]. Pluvinage et al., through CRISPR-Cas9 screening and RNA-seq analysis, identified a marked age-related increase in CD22 expression in microglia, and inhibition of CD22 was shown to improve the clearance of myelin debris, amyloid-β, and α-synuclein fibers. These findings implicate CD22 as a negative regulator of microglial phagocytic activity in the central nervous system, highlighting its potential involvement in the pathogenesis of age-related neurodegenerative disorders [85]. The work of Aires et al. also suggests that the expression of CD22 in aging microglia may potentially impact the occurrence of neurodegenerative lesions. The authors’ findings indicate that CD22 expression may serve as a significant marker linked to macrophages’ phagocytic function and aging.

sMACs is also tied to a decrease in the expression of TLRs and MHC-II. TLRs can recognize conserved elements in pathogens, activating immune cells, such as macrophages and dendritic cells [25]. The levels of TLRs expressed in splenic and peritoneal macrophages from aged mice has been shown to be significantly reduced, and following stimulation with TLR ligands, the production of pro-inflammatory factors, such as IL-6 and TNF-α is markedly diminished [26]. Additionally, the interaction between TLRs and adenosine A receptors plays a pivotal role in regulating macrophages’ secretion of vascular endothelial growth factor (VEGF) and other angiogenic factors. Consequently, the diminished expression of TLRs in macrophages may impair their capacity for effective wound healing [86–88]. Moreover, macrophages present antigenic peptides via MHC-II molecules to helper T cells, initiating adaptive immune responses. Numerous studies have indicated that aging reduces macrophages’ ability to express MHC-II genes in response to IFN-γ stimulation [89–91].

The respiratory capacity of macrophages also declines with age. Enhanced p38 mitogen-activated protein kinase (MAPK) activity in sMACs inhibits T-cell immunoglobulin mucin protein 4 (TIM4) receptor’s expression, impairing its ability to phagocytose apoptotic bodies. This attenuation in clearance mechanisms can hinder the resolution of acute inflammation, potentially triggering the pathogenesis of chronic inflammatory diseases, including Alzheimer’s disease and atherosclerosis [92]. Moreover, sMACs exhibits activation of the kynurenine pathway (KP), which suppresses quinolinic acid phosphoribosyl transferase (QPRT) activity and impairs nicotinamide adenine dinucleotide (NAD) synthesis. This leads to a reduction in mitochondrial respiration capacity and fosters a pro-inflammatory phenotype [93–96], further exacerbating the depletion of tissue NAD levels through increased activity of the NAD-consuming enzyme CD38 [97]. The above metabolic indicators, the TIM4 receptor, and CD38 may also serve as biomarkers for sMACs.

## Potential molecular regulatory mechanisms of sMACs in shaping the TME

**Fig. 2:**
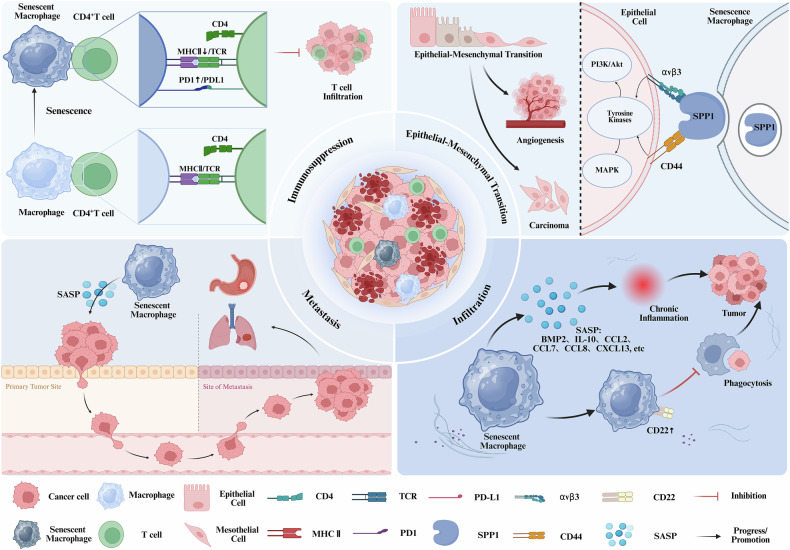
The potential role of senescent macrophages on tumors. (1) Senescent macrophages suppress T cell proliferation through the downregulation of MHC II expression and the upregulation of PD-1 expression. (2) Senescent macrophages promote epithelial-mesenchymal transition (EMT) by increasing SPP1 expression, thereby facilitating tumor growth and angiogenesis. (3) Senescent macrophages’ secretion of senescence-associated secretory phenotype (SASP) factors drives tumor metastasis. (4) Senescent macrophages promote tumorigenesis and progression by upregulating CD22 expression, reducing phagocytic capacity, and inducing chronic inflammation through SASP secretion.

The disordered proliferation of malignant cells is accompanied by the senescence of other cells, suggesting that inhibition and restoration may be equally important in treating tumors. Lelinh et al. conducted experiments demonstrating that mesothelioma exhibited a more rapid proliferation rate in aged mice, accompanied by a higher density of infiltrating TAMs within the tumors [98]. Daniella et al. analyzed data from The Cancer Genome Atlas for prostate adenocarcinoma (TCGA-PRAD) and found that the expression levels of macrophage-related genes, including CD163 and VSIG4, were linked to tumor recurrence, and were significantly higher in prostate cancer tissues from older patients [99]. Additionally, Li et al. presented that in tumor tissues of older patients, the infiltration rate of CD68^+^ CD206^+^ cells was higher than that in younger patients, and sMACs co-cultured with tumor cells could enhance the size of tumors [100]. An increasing body of evidence indicates that sMACs are prevalent in tumors, where they support a pro-tumor microenvironment phenotype by activating multiple molecular pathways (Fig. 2).

### Programmed death-ligand 1 (PD-L1)

Julia et al. conducted a single-cell analysis of murine lung tissues and found a significant increase in alveolar macrophages in aged mice. In a model of chronic pneumonia, the stability of programmed death-ligand 1 (PD-L1) protein was increased in sMACs in lung epithelial cells; the protein degradation was slowed via P16-mediated inhibition of CDK4/6 pathways [101]. In the TME, both tumor cells and non-tumor cells express PD-L1. While this mechanism maintains immune system equilibrium in healthy physiological states, it facilitates immune escape by tumor cells in the TME, consequently supporting their proliferation and metastasis [102–105]. In recent years, blocking the PD-L1/PD-1 pathway has shown significant anti-tumor effects in advanced cancer patients; this pathway has become a direction of intense development of new immune checkpoint blockade therapies and their combinations. Several antibodies targeting PD-1/PD-L1 are undergoing clinical trials, representing a crucial component in targeted therapy [106, 107]. However, many patients are resistant to this treatment, particularly those with solid tumors, which may be primarily related to irreversible T cell exhaustion, dysfunctional MHC expression and activity, along with resistance to IFN-γ signaling [108]. Since macrophages represent the predominant population of innate immune cells within the TME, and are closely linked to tumorigenesis and chronic inflammation, targeting PD-L1 expression in sMACs may provide innovative strategies with promising clinical outcomes in tumor immunotherapy.

### Secreted phosphoprotein 1 (SPP1)

Single-cell RNA sequencing of aged murine skeletal muscle by Bi et al. identified an SPP1^high^ macrophage subset enriched in senescent tissue. This population showed elevated expression of senescence markers and activation of VEGF and lipid metabolism pathways, indicating a potential role in immunometabolic remodeling and tissue homeostasis during aging [109]. Yu et al. constructed an aging-related prognostic model for colorectal cancer (CRC) based on TCGA and GEO databases. The authors found that patients in the high-risk group had an abundance of macrophages in their tumor tissues, exhibiting immune suppression characteristics. Data analysis further identified a distinct subpopulation of macrophages with elevated SPP1 expression in high-grade tumors, the macrophages concurrently displayed pronounced features of the SASP. These macrophages were surrounded by senescent tumor cells, indicating a potential correlation with poor prognosis linked to cellular senescence. SPP1, or osteopontin, is a multifunctional secreted phosphoglycoprotein primarily expressed in immune cells like macrophages and dendritic cells, though it can also be highly expressed in certain tumor cells. Extensive research has demonstrated that the expression levels of SPP1 in TAMs are positively correlated with tumorigenesis, invasion, metastasis progression, and the exacerbation of adverse clinical prognoses. Furthermore, the metabolic characteristics of SPP1^+^ macrophages and the functional properties of the ECM exhibit marked alterations [110–112]. SPP1 induces the activation of the PI3K/Akt and MAPK pathways through its interaction with integrin αvβ3 and CD44, thereby facilitating EMT, and epigenetic modifications and contributing to the development of drug resistance. Therefore, SPP1 is considered a potential novel biomarker for TAMs and may even become an important therapeutic target [113–115]. Despite the extensive research on the critical role of SPP1 in TAMs, its relationship with sMACs requires additional experimental exploration.

### C-X-C chemokine receptor 1 (CXCR1)

Luis et al. established a kirsten ratsarcoma viral oncogene homolog (KRAS) mutation-driven lung adenocarcinoma mouse model to identify a population of p16^+^CXCR1^+^aging macrophages within tumor tissue through single-cell sequencing analysis. This study identified p16⁺CXCR1⁺ sMACs as key mediators of immune suppression by secreting IL-8 and upregulating PD-L1, thereby promoting a tumor immune-cold microenvironment through T cell inhibition. Notably, the depletion of this senescence macrophage population significantly slowed the progression of lung adenocarcinoma, indicating that these p16^+^CXCR1^+^ macrophages are crucial in altering the TME and thus contributing to tumorigenesis. This study represents the first to propose CXCR1 as a potential biomarker for macrophage senescence in the context of lung adenocarcinoma [116]. CXCR1 is a seven-transmembrane G protein-coupled receptor (GPCR) that belongs to the GPCR superfamily. It primarily transmits inflammatory signals and regulates immune responses by recruiting and activating leukocytes [117]. A growing body of research has demonstrated that CXCL8 and its homologous receptors, CXCR1 and C-X-C chemokine receptor 2 (CXCR2), play significant roles in the occurrence and progression of various cancers, including breast cancer, prostate cancer, lung cancer, CRC, and melanoma. These chemokines and their receptors regulate the proliferation, invasion, and migration capacity of tumor cells via autocrine or paracrine mechanisms [118, 119]. In prostate cancer, the CXCL8/CXCR1 axis primarily drives tumor cell proliferation and demonstrates a significant association with unfavorable clinical outcomes [120]. Additionally, CXCR1 has been shown to be constitutively expressed in all cases of melanoma [121]. Thus, targeting CXCR1 may represent a novel strategy for treating various malignancies [122].

### Receptor for activated C kinase 1 (RACK1)

RACK1, a key member of the RACK family, functions as a multifunctional scaffold protein that orchestrates diverse signaling pathways involved in cell cycle regulation, proliferation, migration, and senescence [123]. Studies have shown that RACK1 promotes the ubiquitination and degradation of p53 by recruiting MDM2 through its interaction with FGFR, thereby suppressing p53-dependent senescence [124]. It can also inhibit p21^Cip1^ expression by binding to Smad3, blocking p21-induced growth arrest [125]. These mechanisms, validated in lung cancer cells and neural stem cells, may similarly regulate macrophage proliferation and senescence.

In sMACs, RACK1 expression is closely associated with immune function. With aging, RACK1 levels decline in alveolar macrophages, impairing cytokine secretion such as tumor necrosis factor alpha upon LPS stimulation, and reducing the release of SASP components like CCL2, CCL5, and IL-6 [126]. This downregulation may contribute to immunosenescence and the shift of macrophages toward a tumor-promoting phenotype.

Moreover, decreased RACK1 expression has been associated with tumor progression across multiple cancers. For instance, Helicobacter pylori infection suppresses RACK1 via the integrin β1/NF-κB pathway in gastric cancer, facilitating inflammatory signaling and tumorigenesis [127]. In colorectal and pancreatic carcinoma, low RACK1 levels correlate with increased invasiveness and chemoresistance [128]. Together, these findings underscore RACK1’s dual role in maintaining immune homeostasis and suppressing tumorigenesis, suggesting its potential as a therapeutic target in sMACs-driven pathologies.

### Lymphatic endothelial hyaluronan receptor 1 (LYVE1)

Linda et al. conducted single-cell RNA sequencing (scRNA-seq) analysis of skeletal muscle and found that the number of LYVE1 macrophages was greater than that of LYVE1^+^ macrophages in aged skeletal muscle. Moreover, the expression levels of pro-inflammatory markers (such as S100a8 and S100a9 mRNA) and senescence-associated markers (such as GPNMB and SPP1 mRNA) exhibited a significant positive correlation with the increased LYVE1^-^ macrophages. This suggests that LYVE1 may be a key marker for distinguishing sMACs [129]. LYVE1 is a CD44 homolog encoded by the LYVE1 gene [130, 131]. In certain cancers, LYVE1 expression levels are significantly elevated, and accumulating evidence implicates it in the initiation and metastatic progression of the primary tumor. In particular, LYVE1 secreted by macrophages has been shown to inhibit melanoma cell proliferation and foster lymphangiogenesis, thereby providing further evidence for the potential role of LYVE1 aging macrophages in the pathogenesis and progression of malignancies [132, 133].

### Aldo-Keto reductase family 1 member B1 (AKR1B1)

Zhang et al. analyzed TCGA gastric cancer data and identified five genes related to aging within the gastric cancer TME, among which AKR1B1 was notably included [134]. Subsequently, Jiang et al. used the CELLAGE and TCGA databases to analyze scRNA-seq markers, identifying 23 common aging-related macrophage genes in bladder cancer patients. The study further confirmed a significant association between the upregulation of AKR1B1 in sMACs and the occurrence and progression of bladder cancer [135]. In vitro experiments showed that hydrogen peroxide-induced sMACs significantly upregulated AKR1B1 expression, enhancing the proliferation and migration of gastric and bladder cancer cells [136]. Aldose-ketose reductase family 1 (AKR1) is a family within the aldose-ketose reductase (AKR) superfamily, and AKR1B1 is one of its subfamilies [137]. The aging-related tumor suppressor gene P53 has been implicated in modulating the expression of AKR1B1, thereby exerting influence over the metastatic characteristics of breast cancer [138]. Multiple studies have confirmed that AKR1B1 is overexpressed in various tumors. For instance, in lung cancer, suppression of AKR1B1 expression has been shown to restore tumor sensitivity to EGFR tyrosine kinase inhibitors (TKIs) [139]. Additionally, in endometrial and ovarian cancers, AKR1B1 expression levels are significantly correlated with cellular oxidative stress and prognosis. In macrophages, AKR1B1 upregulation supports ROS-NF-κB signaling, promoting SASP factor secretion and potentially enhancing the pro-tumorigenic effects of sMACs [140]. Nonetheless, the precise association between AKR1B1 and aging macrophages necessitates further investigation and empirical validation to elucidate the underlying mechanisms.

## Novel strategies for cancer therapy targeting sMACs

### Senolytics

**Fig. 3:**
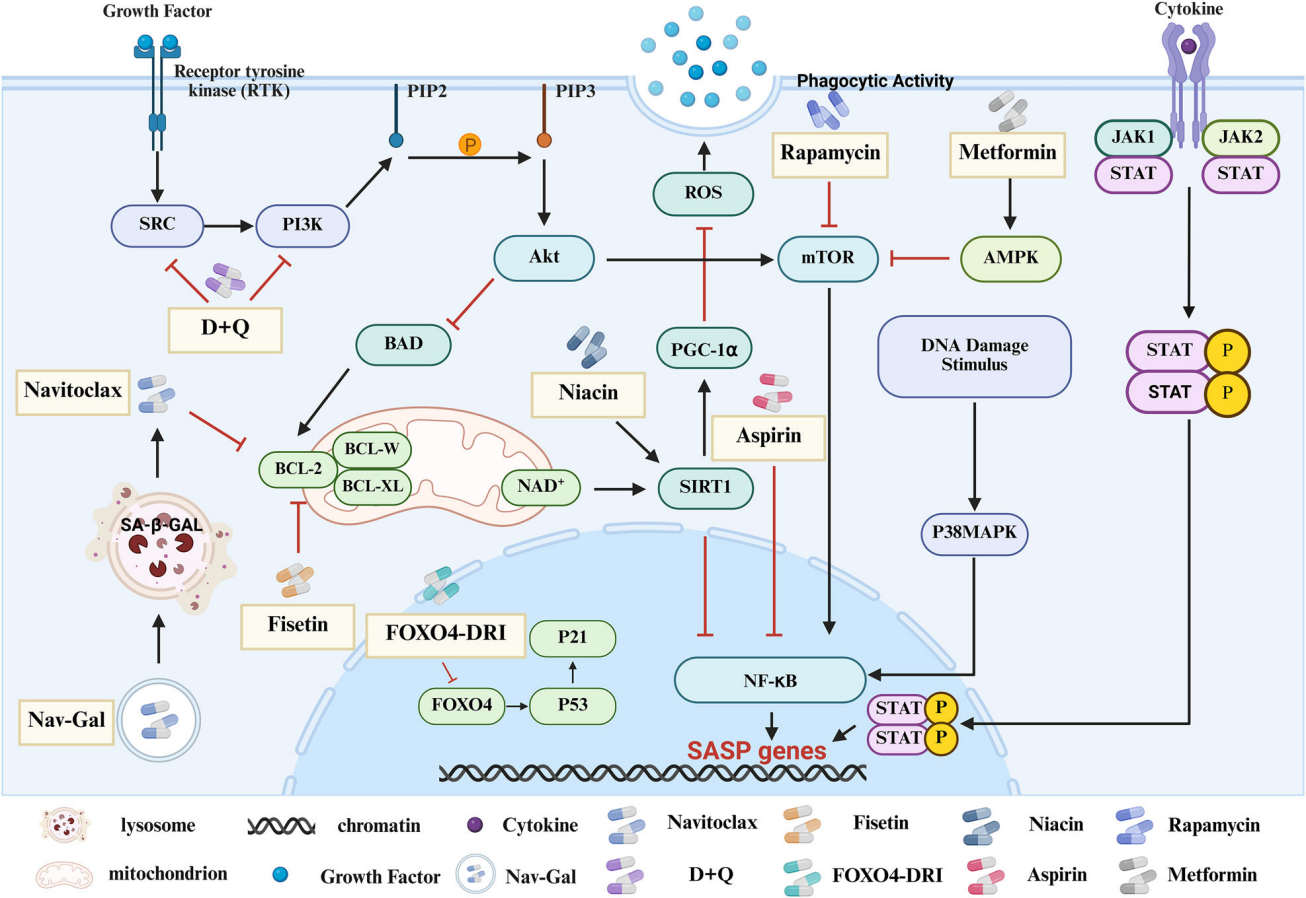
Senolytics and senomorphics eliminate senescent cells or suppress the SASP via multiple mechanisms. D + Q, a tyrosine kinase inhibitor, induces apoptosis in senescent cells; Navitoclax and Fisetin promote apoptosis primarily through inhibition of Bcl-2 family proteins; FOXO4-DRI binds to FOXO4, disrupting its interaction with p53 to induce cell death; Niacin facilitates clearance by enhancing phagocytic activity; Rapamycin, Aspirin, and Metformin suppress SASP release by inhibiting NF-κB-related signaling pathways.

The increasing evidence highlighting the close involvement of senescent cells in cancer pathogenesis necessitates exploring effective strategies for their targeted elimination (Fig. 3). Although senescent cells secrete pro-apoptotic SASP factors, these cells have been shown to resist apoptosis. It is hypothesized that senescent cells may evade clearance through protective anti-apoptotic pathways [141–143]. Bioinformatics analyses of proteomic and transcriptomic data from senescent and non-senescent cells have identified several senescent cell anti-apoptotic pathways (SCAPs), which mean survival pathways upregulated in senescent cells that help protect them from apoptosis, including the PI3Kδ/AKT, BCL-2/BCL-xL/BCL-W, and the p53/FOXO4a/p21/serpin [plasminogen activator inhibitor 1 and 2 (PAI-1, PAI-2)] pathways [142, 144]. Following the integration of SCAPs, and screening of drug libraries, numerous senolytic drugs have been identified, many of which are anticancer agents. These include tyrosine kinase inhibitors, such as dasatinib and quercetin (D + Q), and BCL-2 family inhibitors like venetoclax. These compounds have demonstrated promising effects in various studies [145–148].

Currently, therapeutic strategies targeting sMACs are limited and still in the early stages of development. Table 1 presents various potential pharmacological agents against senescent macrophages. Navitoclax (ABT-263), in combination with D + Q, has been shown to alleviate pulmonary inflammation induced by COVID-19 in mouse models, through the targeted clearance of senescent cells, including macrophages [149]. Navitoclax, a selective inhibitor targeting the anti-apoptotic protein BCL-2, has been shown to improve the phagocytic capacity of macrophages by upregulating the Trem-2 receptor. Furthermore, the drug induces berlin-1-dependent autophagy, significantly reducing sepsis in aged mice [150]. The tyrosine kinase inhibitor D + Q, the earliest identified senolytic drug, effectively eliminates most senescent cells, including macrophages. Medical research has extensively examined the formulation to explore its potential therapeutic applications [109, 151]. Fisetin, a BCL-2 inhibitor, has demonstrated the ability to suppress LPS-induced macrophage activation. Furthermore, Fisetin inhibits ox-LDL-induced senescence in RAW264.7 macrophages via the CKIP-1/REGγ signaling pathway [152, 153]. Nicotinic acid (vitamin B3) and its metabolites have been shown to significantly suppress the generation of ROS, NO, and nitric oxide synthase 2 (NOS2) in LPS-stimulated human macrophages. These compounds also enhance the phagocytic activity of microglia for myelin by upregulating CD36 expression in macrophages [154, 155]. Ganciclovir and D + Q have been shown to restore the phagocytic capacity of irradiated, splenic-derived macrophages by eliminating senescent cells [156]. Cystamine treatment of cultured macrophages has been shown to significantly decrease the production of ROS. Moreover, the compound attenuates macrophage senescence by inhibiting lysosomal oxidation of low-density lipoprotein (LDL) in human macrophages. Additionally, cystamine inhibits the production of TNF-α, IL-6, and MCP-1 by sMACs, thereby markedly reducing the incidence of atherosclerosis in aged mice [157–160]. Selective induction of apoptosis in senescent-like CAFs using the FOXO4-p53 interfering peptide, FOXO4-DRI, enhances the sensitivity of non-small cell lung cancer to radiotherapy and reduces radiation-induced pulmonary fibrosis. FOXO4-DR peptide avoids direct inhibition of BCL-XL, thereby sparing platelets from toxicity [161]. While some anti-aging drugs have already been applied in clinical settings, further research is needed to explore their effects on sMACs and the impact of clearing these cells on tumor progression. Additionally, it is essential to assess the implications of senolytic-mediated clearance of sMACs on tumor biology and progression.

**Table 1:** Potential therapeutic agents for senescent macrophages and their mechanisms.

Category	Drugs	Mechanism	Clinical Trials	Citations
Senolytics	Navitoclax	Increases the phagocytic ability of macrophages by upregulating the Trem-2 receptor and blocking the interaction between BCL-2 and beclin-1, thereby inducing beclin-1-dependent autophagy.		[150]
Senolytics	Dasastinib and quercetin (D + Q)	Inhibits multiple tyrosine kinases (such as Src, ABL, c-Kit, etc.) and induces apoptosis.	NCT04946383 NCT05422885	[109]
Senolytics	Fisetin	Inhibits ox-LDL-induced senescence in RAW264.7 macrophages through the CKIP-1/REGγ pathway. Inhibiting Bcl-2 family proteins promotes apoptosis of senescent cells.	NCT03675724 NCT06399809 NCT06133634 NCT03430037	[153]
Senolytics	Niacin (Vitamin B3)	Upregulates the expression of CD36 in macrophages, enhancing the phagocytic activity of microglia for myelin.	NCT06592859 NCT02921659 NCT06208527	[155]
Senolytics	Ganciclovir	Eliminates senescent cells, including senescent macrophages.		[156]
Senolytics	Cysteamine	Inhibits the lysosomal oxidation of LDL in human macrophages, reducing macrophage senescence.		[158, 159]
Senolytics	FOXO4-DRI	FOXO4-DRI can bind to FOXO4, prevent its interaction with p53, release p53, and promote apoptosis.		[161]
Senomorphics	Rapamycin	Suppresses pro-inflammatory SASP production at the translational level by downregulating NF-κB activity.	NCT06550271 NCT04742777 NCT02874924 NCT04994561	[164]
Senomorphics	Aspirin	Blocks NF-κB to reduce inflammatory cytokines.		[18]
Senomorphics	Metformin	Activates AMPK to suppress mTOR and lower SASP secretion.	NCT02432287 NCT02308228 NCT03309007	[165]

### Senomorphics

In addition to senolytics, a class of therapeutic strategies known as senomorphics has recently garnered attention. Senomorphics aim to modulate the expression of the SASP to reduce its detrimental impact on the tissue microenvironment, without significantly affecting cell cycle arrest itself [162]. These drugs primarily act by intervening in key signaling pathways closely associated with SASP regulation, including p38 MAPK, PI3K/Akt, mTOR, JAK/STAT, and NF-κB. These pathways play essential roles in the induction, maintenance, and amplification of SASP, making them central targets for senomorphic drug intervention [163].

One of the classic senomorphic agents, rapamycin—an inhibitor of the mTOR—has been widely employed in studies targeting cellular senescence. Rapamycin suppresses pro-inflammatory SASP production at the translational level by downregulating NF-κB activity and has been shown to limit the tumor-promoting effects of senescent fibroblasts within the microenvironment of cancers such as prostate cancer [164]. Aspirin blocks NF-κB to reduce inflammatory cytokines, while metformin activates AMPK to suppress mTOR and lower SASP secretion. Together, these agents help alleviate the inflammatory microenvironment associated with cellular senescence [18, 165].

## Challenges and limitations of sMACs

### Multifaceted immune regulation of the SASP-related sMACs

SASP, the primary substance secreted by aging cells, comprises a series of inflammatory and interleukin factors, suggesting it plays a more complex and multifaceted role. For example, SASP secreted by precancerous hepatocytes after senescence activates the NF-κB signaling pathway, induces the expression of GM-CSF, Arg1, and IL-6, and significantly enhances CD4⁺ T cell-mediated “senescence surveillance” [166, 167]. In the early stage of HCC, these senescent cells can also recruit CCR2⁺ myeloid cells by secreting CCL2 to eliminate potentially malignant cells and delay tumor formation [168]. On the other hand, in prostate cancer, chemokines and cytokines secreted by senescent cells recruit immunosuppressive MDSCs to weaken anti-tumor immunity in the TME [168]. IL-6, IL-8, and MMPs contained in SASP can significantly promote the invasion and metastasis of pancreatic cancer cells [169]. These findings not only suggest that SASP-related sMACs may have individual differences in different histopathologies or tumor types, but also may play multifaceted immune regulatory roles in the occurrence and development of tumors.

In addition, the senescence of other cells in the TME, including stromal cells, and the background of therapeutic senescence due to chemotherapy and radiotherapy interventions, together enhance the complexity of aging phenotypes within the TME. It remains to be elucidated about the synergistic or exclusive relationship between myeloid-originated sMACs and intratumoral induced sMACs, as well as the relationship between “intratumoral total aging pattern” and immune regulation.

### Appropriate sMACs research models to establish causality

The role of sMACs in tumors and chronic diseases has attracted much attention, but the current studies on their functions mostly focus on the relationship between expression levels. For example, single-cell RNA sequencing identified a macrophage subset with high expression of p16, or bulk-TCGA transcriptome data analysis showed that sMACs infiltration was positively correlated with poor prognosis of cancers [116, 135]. The development of a specific and controllable sMACs research model is essential for further research to establish a clear causal relationship.

In the construction of sMACs cells in vitro, other stimulation methods of senescent cells can be used for reference, including cell senescence, which can be induced by radiation, chemical induction, or CRISPR-edited p16 or other senescence promoter genes [170]. For example, Kim et al. used ionizing radiation and doxorubicin to induce senescence of human umbilical vein endothelial cells and found that CXCL11 secreted by them promoted breast cancer invasion, and blocking the CXCL11/CXCR3 pathway could attenuate this effect [171].

In vivo studies, p16Ink4a knockout mice (p16-KO) are often used to analyze senescence-related signals, but p16 deficiency limits the normal occurrence of aging and affects functional studies [172]. Therefore, INK-ATTAC and p16-3MR can induce senescent cell depletion in mouse models by specifically eliminating p16-positive senescent cells, which can effectively evaluate their causal role in tissue homeostasis, chronic inflammation, and disease [173]. Recently, Jean-Philippe et al. revealed the role of aging myeloid cells in the development of lung cancer by transplanting the bone marrow of aging mice into young mice, which provided a new idea for the construction of sMACs or other myeloid cell aging models [80].

### Challenges in sMACs-specific targeting

Current senolytic and senomorphic strategies face several limitations, with off-target effects being among the most critical concerns [174, 175]. For example, while the Bcl-2 inhibitor Navitoclax exhibits significant senolytic activity in clearing senescent cells, its clinical application is limited by hematological toxicity, primarily including neutropenia and thrombocytopenia [176, 177]. To mitigate these side effects, various strategies have been explored, such as intermittent dosing regimens or individualized dose adjustments to promote platelet recovery. Combination therapy has also been proven effective in reducing monotherapy exposure, such as combining Navitoclax with ruxolitinib or rituximab, which effectively lowers the required dose while maintaining therapeutic efficacy [178]. Furthermore, alternative therapeutic approaches are under development, including the design of PROTAC molecules that specifically degrade Bcl-XL, thus avoiding direct effects on platelets. Additionally, as mentioned earlier, FOXO4-DR peptides represent a novel class of senolytic drugs that protect platelets by not inhibiting Bcl-XL, thereby reducing hematologic toxicity [161]. Recently, a β-galactosidase-triggered galactose-conjugated nanodrug system (nav-Gal) has been developed, which allows for the selective release of Navitoclax in senescent cells, significantly enhancing therapeutic targeting and minimizing systemic toxicity [175].

In addition, senescent cells (positive for p16INK4a and SA-β-Gal) detected in osteoarthritis (OA) synovium may be derived from both fibroblasts and macrophages [179]. After intraperitoneal injection of senescent cells into mice, the researchers found that F4/80-positive macrophages were predominantly clustered around them, and they also expressed p16 and SA-β-Gal [56]. Moreover, the differences in sMACs across species (e.g., murine vs. human sMAC phenotypes) are also unknown. The above factors together increase the difficulty of targeted clearance of sMACs in clinical applications.

## Conclusion

**Fig. 4:**
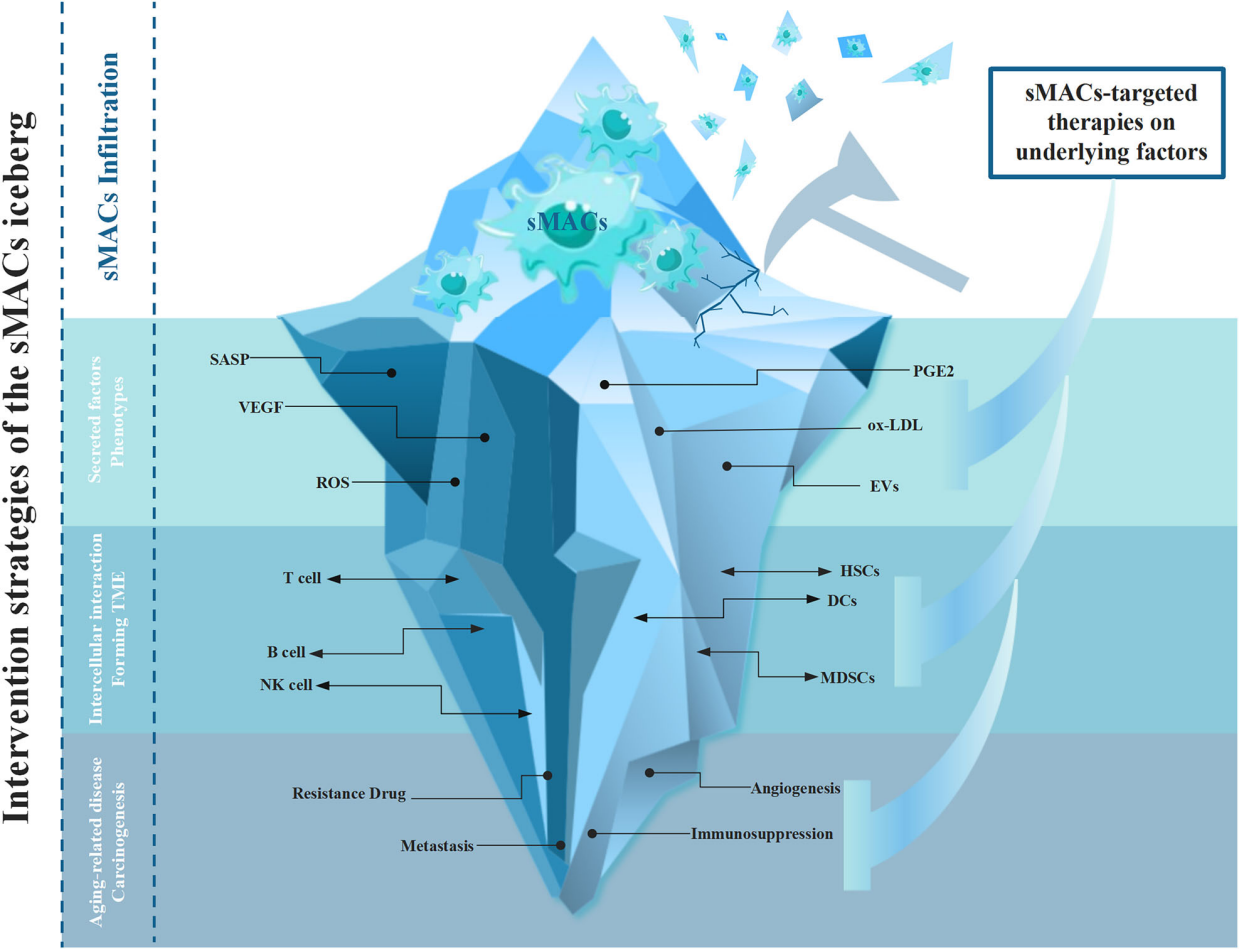
Intervention strategies of the sMACs iceberg. The infiltration of sMACs in tissues is like an iceberg above the water, which can be observed and detected. The correlation between the sMACs and the phenotypes and secreted factors, with the intercellular interaction and the formation of TME, with the aging-related diseases and carcinogenesis, like the underwater iceberg, still needs to be researched. The underlying factors at different levels should also be considered in the design of future clinical trials of sMACs-targeted therapies.

With the continuous progress of research technology and the wide application of single-cell multi-omics technology, sMACs have been found in different tumors, but there is still a lack of research on the causality at the mechanistic level. The sMACs contain a series of secreted factors and molecular biomarkers, which not only expand our understanding of the multifaceted functions of sMACs in immune regulation, but also raise questions about how sMACs participate in the construction of balance in tumors (Fig. 4). Although a variety of anti-aging drugs have entered clinical trials, whether these drugs are effective in sMACs remains to be evaluated. Given that specific targets of sMACs have not yet been developed, finding strategies similar to PD1/PDL1 intervention to reverse T cell exhaustion may be a potential direction for its future clinical applications.
